# Sleep quality among military personnel exposed to high altitude: a cross-sectional study of oxygen supplementation and altitude transition

**DOI:** 10.3389/fpubh.2026.1854316

**Published:** 2026-06-15

**Authors:** Qian Yang, Yi Liu, Chen Xu, Fugui Li, Xiaofan Yan, Xiaoli Zong, Li Peng

**Affiliations:** Department of Military Psychology, Faculty of Medical Psychology, Army Medical University, Chongqing, China

**Keywords:** altitude transition, high-altitude exposure, hypoxia, military personnel, oxygen supplementation, sleep quality

## Abstract

**Background:**

Sleep disturbances are common among military personnel stationed in high-altitude environments and may have important implications for health and operational functioning. This study aimed to investigate the effects of cumulative high-altitude exposure on sleep quality and to examine the roles of oxygen supplementation and frequency of returning to low-altitude areas.

**Methods:**

A cross-sectional survey was conducted among 1,316 military personnel stationed in high-altitude regions. Sleep quality was assessed using the Pittsburgh Sleep Quality Index (PSQI), with higher scores indicating poorer sleep quality. Participants also reported demographic characteristics, cumulative duration of high-altitude residence, oxygen supplementation frequency, oxygen supplementation duration per session, and frequency of returning to low-altitude areas. Bivariate correlation analyses and hierarchical regression analyses were conducted to examine the associations and interaction effects among these variables.

**Results:**

Sleep difficulties were positively correlated with cumulative high-altitude residence duration (*r* = 0.13, *p* < 0.001) and frequency of returning to low-altitude areas (*r* = 0.07, *p* < 0.001), and negatively correlated with oxygen supplementation frequency (*r* = −0.20, *p* < 0.001) and oxygen supplementation duration per session (*r* = −0.12, *p* < 0.001). A significant three-way interaction was observed among cumulative high-altitude residence duration, oxygen supplementation, and frequency of returning to low-altitude areas. Specifically, cumulative high-altitude residence duration was significantly associated with greater sleep difficulties (*b* = 0.41, *p* = 0.003) under the combined condition of shorter oxygen supplementation sessions and more frequent returns to low-altitude areas.

**Conclusion:**

These findings suggest that optimizing oxygen supplementation strategies and managing altitude-transition frequency may help reduce sleep disturbances and improve overall health outcomes among military personnel in high-altitude environments.

## 1 Introduction

High-altitude regions are characterized by low oxygen partial pressure, cold climate, dry air, and intense ultraviolet radiation. These environmental stressors can induce a spectrum of physiological and psychological disturbances including headaches, insomnia, neurological dysfunction, and even life-threatening pathologies like high-altitude cerebral edema and pulmonary edema (1–3). Among these conditions, sleep disturbance is one of the most prevalent complaints, which not only occurs during acute high-altitude exposure (4), but also worsens progressively with prolonged residency at high altitudes (5). Emerging evidence suggests that chronic sleep disruption at high altitudes can lead to neurocognitive deficits (6), impair immune function (7), and accelerate cellular aging through telomere shortening (8, 9).

For military personnel deployed to high-altitude regions, the consequences of poor sleep quality are particularly pronounced. Impaired sleep has been linked to a 3- to 4-fold increase in tactical errors during military exercises (10) and a significantly elevated risk of altitude-related accidents (11). Furthermore, sustained sleep impairment severely compromises long-term health outcomes and quality of life (12). High-altitude military deployments expose personnel to extreme physical and psychological stressors, making sleep quality a critical determinant of operational effectiveness, far beyond a matter of personal comfort. Empirical studies have demonstrated that inadequate sleep under high-altitude conditions impairs physiological function, enhanced cognitive performance, and emotional stability, while also exacerbating physiological strain and hindering recovery processes (13, 14). In short-term deployments, acute mountain sickness (AMS), dyspnea, and insomnia are prevalent; over the long term, sleep quality tends to deteriorate with age, length of military service, and cumulative high-altitude exposure (15). These findings underscore the urgent need to identify modifiable factors that affect sleep quality among high-altitude military personnel.

Hypoxia is a key physiological challenge that impairs sleep quality at high altitudes. Chronic exposure to low oxygen levels disrupts sleep architecture by increasing the proportion of light non-rapid eye movement (NREM) sleep, mainly stages N1 and N2, while reducing deep NREM sleep, namely slow-wave sleep or stage N3, and rapid eye movement (REM) sleep, leading to difficulties in falling asleep, fragmented sleep, and early morning awakenings (6). Oxygen supplementation has been widely recognized as the most direct and effective intervention for correcting hypoxia. Evidence from high-altitude populations shows that long-term oxygen supplementation significantly improves sleep respiratory rhythm, sleep quality and cardiovascular functions (16). In clinical populations such as patients with obstructive sleep apnea (OSA), nocturnal oxygen supplementation reduces the apnea-hypopnea index (AHI) (17). For individuals experiencing acute high-altitude exposure, short-term nocturnal oxygen supplementation can alleviate nocturnal hypoxemia and periodic breathing disturbances, thereby improving sleep quality (18). Notably, intermittent oxygen supplementation (e.g., limited to the first half of sleep) may be insufficient to maintain stable blood oxygen levels throughout the night (19), whereas continuous oxygen supplementation more effectively stabilizes oxygenation (16), reduces respiratory events, and decreases sleep fragmentation. Previous studies have also examined oxygen concentration or oxygen-enrichment levels during nocturnal oxygen supplementation. For instance, prior high-altitude studies have manipulated room-air oxygen enrichment levels to assess their effects on sleep architecture, nocturnal oxygenation, and sleep quality (20). However, in field-based military settings, exact inspired oxygen concentration is often difficult to monitor continuously. Therefore, the present study focused on two more feasible and reliably recorded indicators of oxygen use: supplementation frequency and duration. Despite growing evidence supporting the benefits of oxygen supplementation, previous studies lacked a detailed investigation into the differential impacts of varying supplementation frequencies and durations, particularly in relation to individuals’ cumulative high-altitude residence. It remains unexplored how these supplementation variables interact with high-altitude exposure to influence sleep quality.

In addition to oxygen supplementation patterns, frequent transitions between high- and low-altitude areas may represent another key factor affecting sleep quality. Repeated environmental shifts demand continuous physiological readaptation, potentially heightening stress responses and disrupting homeostasis. Rapid ascent to high-altitude regions may increase the risk of AMS, characterized by symptoms such as headache, nausea, and fatigue (21). Conversely, upon returning to low-altitude areas, individuals may experience high-altitude de-acclimatization syndrome (HADAS), manifesting as fatigue, sleep disturbances, and cognitive impairment (22, 23). Frequent transitions between altitudes may prolong or exacerbate these symptoms. Additionally, psychological and social stress arising from repeated family reunions and separations during these transitions may exacerbate emotional distress, further impairing sleep quality (24).

Sleep disturbances among military personnel stationed at high altitudes represent an important public health concern, as they may impair physical functioning, cognitive performance, and operational safety (10–12). Despite growing evidence on the role of hypoxia and oxygen supplementation in sleep disturbances at high altitudes (16–19), limited research has examined how cumulative high-altitude exposure interacts with modifiable behavioral and environmental factors in shaping sleep outcomes. Understanding these relationships is essential for developing effective health management strategies for military populations operating in extreme environments. Therefore, this study aimed to examine the combined effects of cumulative high-altitude residence, oxygen supplementation patterns, and frequency of returning to low-altitude areas on sleep quality among military personnel.

## 2 Methods

### 2.1 Participants and procedure

This study adopted a cross-sectional survey design. Participants were military personnel stationed in high-altitude regions. A total of 1,316 military personnel were included in the final analysis, of whom 98.9% were male. Participants were aged 18–45 years, with a mean age of 24.68 ± 3.72 years. Detailed demographic and high-altitude exposure-related characteristics are presented in Table 1.

**Table 1:** Sample characteristics.

Variable	*N* (%) or *M* (SD)
Biological sex	
Male	1,301 (98.9)
Female	15 (1.1)
Age	24.68 (3.72)
Ethnicity	
Han	1,176 (89.4)
Tibetan	14 (1.1)
Others	126 (9.6)
Chronic disease history	
No	1,214 (92.2)
Yes	102 (7.8)
Acute mountain sickness	
No	1,171 (89.0)
Mild	138 (10.5)
Moderate	7 (0.5)
Severe	0
First-time high-altitude exposure	
No	854 (64.9)
Yes	462 (35.1)
Cumulative high-altitude residence	
Less than 7 days	19 (1.4)
7 days–1 month	62 (4.7)
1–6 months	530 (40.3)
6 months–1 year	211 (16)
1–3 years	378 (28.7)
3–5 years	93 (7.1)
5–10 years	18 (1.4)
Over 10 years	5 (0.4)
Altitude of current residence	
2,500–3000 m	17 (1.3)
3,000–4000 m	39 (3.0)
4,000–4500 m	1,099 (83.5)
4,500–5000 m	160 (12.2)
Above 5,000 m	1 (0.1)
Oxygen supplementation frequency	
Never	78 (5.9)
Occasionally	376 (28.6)
1–3 times/week	123 (9.3)
3–5 times/week	139 (10.6)
Daily	600 (45.6)
Oxygen supplementation duration per session	
<5 min	146 (11.1)
5–10 min	265 (20.1)
10–20 min	317 (24.1)
>20 min	588 (44.7)
Frequency of returning to low-altitude areas	
None	636 (48.3)
1–2 times/year	618 (47)
3–5 times/year	40 (3)
6–9 times/year	4 (0.3)
10 + times/year	18 (1.4)

Participants were recruited using cluster sampling at the military unit level. Specifically, military units stationed in high-altitude regions were selected as sampling clusters based on their deployment location, feasibility, and administrative coordination. Within each selected unit, all eligible personnel were invited to participate in the survey. The inclusion criteria were as follows: (1) age 18 years or older; (2) current deployment or residence in a high-altitude region; (3) ability to understand and complete the questionnaire independently; and (4) provision of informed consent. No individual-level random sampling was conducted among those who received the survey link. Therefore, although the sample covered military personnel with different durations of high-altitude residence, oxygen supplementation patterns, and altitude-transition frequencies, the generalizability of the findings should be interpreted primarily in relation to young male military personnel stationed in high-altitude environments.

The survey was administered online between September 12 and October 5, 2024. Before completing the questionnaire, participants were informed of the study purpose, voluntary nature of participation, confidentiality of responses, and their right to withdraw at any time. Participants with incomplete questionnaires or obviously invalid responses were excluded from the analysis. Ethical approval for this study was obtained from the Army Medical University Ethics Committee (2024 No. 18-01).

### 2.2 Measures

#### 2.2.1 General information

Participants self-reported their demographic information and high-altitude exposure-related characteristics using a structured questionnaire developed for this study. The questionnaire was designed based on previous literature concerning high-altitude exposure, hypoxia-related sleep disturbance, oxygen supplementation, altitude acclimatization, and high-altitude de-acclimatization. The full content of the questionnaire is provided in Supplementary Appendix A.

Demographic and high-altitude exposure-related variables are presented in Table 1. Biological sex was coded as 0 = female and 1 = male. Chronic disease history was coded as a binary variable, with 0 indicating no history of chronic diseases and 1 indicating a history of at least one listed chronic disease, such as chronic obstructive pulmonary disease, asthma, obstructive sleep apnea, or hypertension. AMS was assessed using the 2018 Lake Louise Acute Mountain Sickness Score (25). The scale includes four symptoms: headache, gastrointestinal symptoms, fatigue/weakness, and dizziness/light-headedness. Each symptom was rated from 0 to 3, yielding a total score ranging from 0 to 12. A total score ≥3, including at least 1 point for headache, was considered diagnostic for AMS. AMS severity was categorized as mild for scores of 3–5, moderate for scores of 6–9, and severe for scores of 10–12.

First-time exposure to a high-altitude environment was also coded as a binary variable (0 = no, 1 = yes). Cumulative duration of high-altitude residence was categorized into eight levels (1 = *less than 7 days* to 8 = *Over 10 years*). Altitude of current residence was classified into five levels (1 = *2,500–3000 m* to 5 = *Above 5,000 m*).

Oxygen supplementation was assessed in terms of frequency and duration per session. In the present study, oxygen supplementation referred to the use of available oxygen-supply methods in high-altitude settings, such as centralized oxygen supply, oxygen cylinders, oxygen bags, or oxygen concentrators, depending on local conditions. Oxygen supplementation frequency was rated on a 5-point scale (1 = *never* to 5 = *daily*), and duration per session was rated on a 4-point scale (1 = *less than 5 min* to 4 = *more than 20 min*).

Frequency of returning to low-altitude areas was defined as the number of times participants returned to low-altitude areas for more than 3 consecutive days per year. Such returns may occur due to temporary leave, family visits, medical visits, training arrangements, or task-related transfers. This variable was categorized into five levels (1 = *none* to 5 = *over 10 times a year*).

#### 2.2.2 Sleep quality

The 19-item Pittsburgh Sleep Quality Index (PSQI) (26) was used to assess participants’ sleep quality over the past month. The PSQI consists of 7 factors: sleep quality, sleep latency, sleep duration, sleep efficiency, sleep disturbances, hypnotic drug use, and daytime dysfunction. Each component was scored from 0 to 3, with the total score ranging from 0 to 21. Higher scores indicate poorer sleep quality. A total score ≥5 indicates clinically significant sleep difficulties. The PSQI has shown good reliability and validity in prior research (27). In this study, the Cronbach’s *α* was 0.897.

### 2.3 Statistical analysis

SPSS 26.0 was used for statistical analysis. Descriptive statistics were calculated for demographic characteristics, high-altitude exposure-related variables, oxygen supplementation variables, frequency of returning to low-altitude areas, and sleep quality. Spearman’s rank correlation coefficients were used for ordinal variables, and Pearson’s correlation coefficients were used for continuous variables where appropriate. Bivariate correlation analyses were conducted to examine associations among the study variables.

Hierarchical regression analysis was conducted to examine the main effects and interaction effects of cumulative duration of high-altitude residence, oxygen supplementation frequency/duration per session, and frequency of returning to low-altitude areas on sleep quality. Two separate models were tested for assessing the moderating roles of oxygen supplementation frequency and duration per session, respectively. In each model, age, chronic disease history, and first-time high-altitude exposure were included as control variables, as these factors were significantly correlated with sleep quality (see Table 2). Control variables were entered at the first step of regression analysis, followed by main effects (cumulative high-altitude residence duration, oxygen supplementation frequency/duration, frequency of returning to low-altitude areas) at the second step, two-way interactions at the third step, and three-way interactions at the fourth step. When interactions were significant, simple slope analysis was conducted at different levels (mean ± 1 SD) of the moderator to further examine the nature of interaction.

**Table 2:** Descriptive statistics and bivariate correlations among study variables.

Variable	M (SD)	Age	Sex^a^	Chronic disease history^b^	Altitude of current residence	First-time high-altitude exposure^b^	Cumulative high-altitude residence	Oxygen supplementation frequency	Oxygen supplementation duration per session	Frequency of returning to low-altitude areas	Sleep difficulties
Age	24.68 (3.72)	–									
Sex^a^	–	−0.03	–								
Chronic disease history^b^	–	0.20^***^	−0.03	–							
Altitude of current residence	3.07 (0.45)	0.02	0.02	0.01	–						
First-time high-altitude exposure^b^	–	−0.54^***^	0.03	−0.13^***^	−0.02	–					
Cumulative high-altitude residence	3.94 (1.21)	0.40^***^	−0.02	0.16^***^	0.23^***^	−0.53^***^	–				
Oxygen supplementation frequency	3.61 (1.44)	0.02	−0.12^***^	−0.09^***^	−0.10^***^	−0.04	−0.08^**^	–			
Oxygen supplementation duration per session	3.02 (1.05)	0.10^***^	−0.09^**^	−0.02	−0.09^**^	−0.03	−0.05	0.45^***^	–		
Frequency of returning to low-altitude areas	1.59 (0.70)	0.32^***^	−0.02	0.08^**^	0.03	−0.42^***^	0.34^***^	0.00	0.04	–	
Sleep difficulties	3.02 (3.08)	0.22^***^	−0.03	0.20^***^	0.03	−0.12^***^	0.13^***^	−0.20^***^	−0.12^***^	0.07^***^	–

a 0 = Female, 1 = Male.

b 0 = No, 1 = Yes.

** *p* < 0.01, *** *p* < 0.001.

## 3 Results

A total of 1,316 military personnel participated in the study. The majority of participants were males, of Han ethnicity, with no chronic diseases, and resided at altitudes of 4,000–5000 m. Among the participants, 35.1% were first-time residents in high-altitude areas, 40.3% had resided in such regions for a cumulative residence of 1–6 months, and 11.0% met the diagnostic criteria for AMS (see Table 1 for details). Additionally, 26.3% reported clinically significant sleep disturbances (PSQI total scores > = 5).

Means, standard deviations, and correlations among study variables are shown in Table 2. Sleep difficulties were positively correlated with age (*r* = 0.22, *p* < 0.001), chronic disease history (*r* = 0.20, *p* < 0.001), cumulative high-altitude residence duration (*r* = 0.13, *p* < 0.001), and frequency of returning to low-altitude areas (*r* = 0.07, *p* < 0.001). Sleep difficulties were negatively correlated with first-time high-altitude exposure (*r* = −0.12, *p* < 0.001), oxygen supplementation frequency (*r* = −0.20, *p* < 0.001), and oxygen supplementation duration per session (*r* = −0.12, *p* < 0.001). Biological sex and altitude of current residence were not significantly correlated with sleep difficulties.

**Figure 1:**
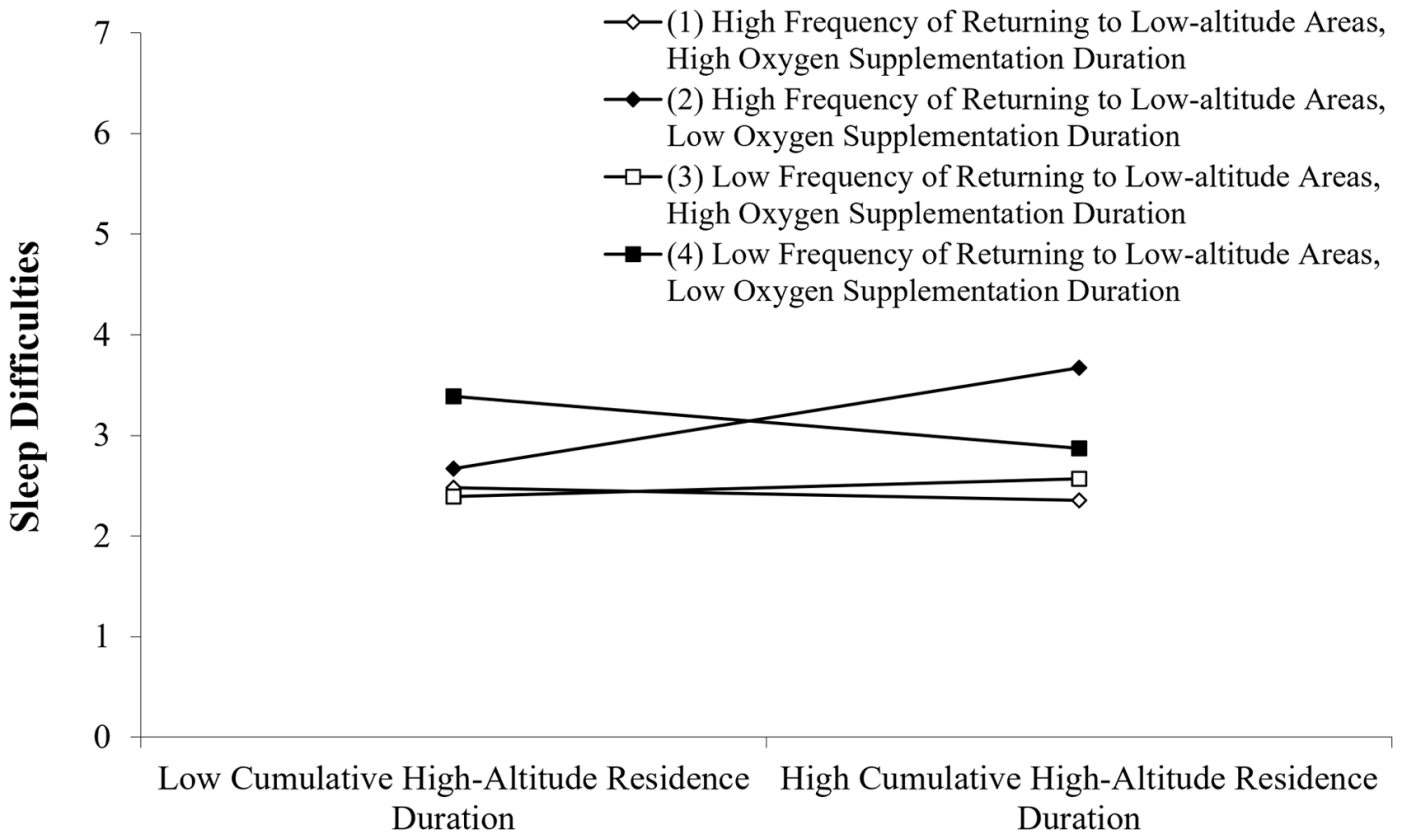
Three-way interaction among cumulative high-altitude residence duration, oxygen supplementation duration, and frequency of returning to low-altitude areas on sleep difficulties. Low and high levels were defined as one standard deviation below and above the sample mean, respectively, following the conventional simple slope analysis approach. Specifically, the low and high conditional values were 1.97 and 4.07 for oxygen supplementation duration per session (*M* = 3.02, SD = 1.05), and 0.89 and 2.29 for frequency of returning to low-altitude areas (*M* = 1.59, SD = 0.70). These values were used only to probe the interaction effect and do not represent empirical grouping cutoffs or clinically established thresholds.

Results of hierarchical regression analysis are shown in Table 3. Both oxygen supplementation frequency and duration per session were negatively associated with sleep difficulties. The main effects of cumulative high-altitude residence duration and frequency of returning to low-altitude areas on sleep difficulties were not significant. No significant interaction effects were observed between oxygen supplementation frequency and either cumulative high-altitude residence duration or frequency of returning to low-altitude areas. However, there was a significant three-way interaction among cumulative high-altitude residence duration, oxygen supplementation duration, and frequency of returning to low-altitude areas. Simple slope analysis showed that cumulative high-altitude residence duration was positively associated with sleep difficulties (*b* = 0.41, *p* = 0.003) only under the combined condition of low levels of oxygen supplementation duration and high levels of frequency of returning to low-altitude areas (Figure 1). This association was not significant under other conditions.

**Table 3:** Main and interactive effects of cumulative high-altitude residence duration, oxygen supplementation frequency/duration, and frequency of returning to low-altitude areas on sleep quality.

Oxygen supplementation frequency and frequency of returning to low-altitude areas as moderators				
Predictor	*B*	*SE*	*β*	*p*
Control variables				
Age	0.16	0.03	0.19	<0.001
Chronic disease history	1.88	0.31	0.16	<0.001
First-time high-altitude exposure	0.04	0.20	0.01	0.858
Main effects				
Cumulative high-altitude residence duration	0.05	0.08	0.02	0.540
Oxygen supplementation frequency	−0.44	0.06	−0.21	<0.001
Frequency of returning to low-altitude areas	0.02	0.13	−0.01	0.889
Two-way interactions				
Cumulative high-altitude residence duration × Oxygen supplementation frequency	−0.06	0.05	−0.03	0.251
Cumulative high-altitude residence duration × Frequency of returning to low-altitude areas	0.11	0.10	0.03	0.405
Oxygen supplementation frequency × Frequency of returning to low-altitude areas	−0.07	0.09	−0.02	0.405
Three-way interactions				
Cumulative high-altitude residence duration × Oxygen supplementation frequency × Frequency of returning to low-altitude areas	−0.11	0.07	−0.05	0.104

Oxygen supplementation duration and frequency of returning to low-altitude areas as moderators				
Predictor	*B*	*SE*	*β*	*p*
Control variables				
Age	0.16	0.03	0.19	<0.001
Chronic disease history	1.88	0.31	0.16	<0.001
First-time high-altitude exposure	0.04	0.20	0.01	0.858
Main effects				
Cumulative high-altitude residence duration	0.08	0.08	0.03	0.335
Oxygen supplementation duration	−0.41	0.08	−0.14	<0.001
Frequency of returning to low-altitude areas	0.002	0.13	0.00	0.990
Two-way interactions				
Cumulative high-altitude residence duration × Oxygen Supplementation duration	−0.07	0.07	−0.03	0.298
Cumulative high-altitude residence duration × Frequency of returning to low-altitude areas	0.15	0.10	0.04	0.132
Oxygen supplementation duration × Frequency of returning to low-altitude areas	0.001	0.12	0.00	0.991
Three-way interactions				
Cumulative high-altitude residence duration × Oxygen supplementation duration × Frequency of returning to low-altitude areas	−0.26	0.09	−0.08	0.004

## 4 Discussion

This study provides evidence that sleep disturbances among military personnel in high-altitude environments are influenced not only by cumulative exposure but also by modifiable behavioral and environmental factors. These findings are broadly consistent with previous research showing that high-altitude conditions are associated with sleep disturbances (4–6) and that oxygen supplementation may improve sleep-related outcomes (16). Our results extend this literature by showing that the effects of cumulative exposure depend on altitude-transition patterns and supplementation behaviors. Specifically, whereas previous studies have mainly focused on acute high-altitude exposure, direct hypoxia-related sleep disturbance, or the general effects of oxygen supplementation, the present study simultaneously considered cumulative high-altitude residence, oxygen supplementation frequency and duration, and repeated transitions between high- and low-altitude areas. This provides a more field-based understanding of sleep quality among military personnel who may experience repeated acclimatization and de-acclimatization during long-term high-altitude deployment.

Consistent with prior research (28), more frequent oxygen supplementation and longer duration per session were associated with fewer sleep difficulties in high-altitude residents. Oxygen supplementation may increase blood oxygen saturation, reduce sleep-related breathing disorders, thereby supporting more stable and restorative sleep (29, 30). A previous study has shown that long-term oxygen therapy significantly improves sleep efficiency, REM sleep duration, and deep sleep stage maintenance, while concurrently reducing nocturnal arousal events induced by central sleep apnea in high-altitude populations (16). This study extends these findings by demonstrating that both the frequency and the duration of oxygen supplementation sessions play significant roles in shaping sleep quality among military personnel. However, because exact oxygen concentration and oxygen flow rate were not measured, the present findings should be interpreted as evidence concerning oxygen supplementation patterns rather than precise oxygen-dose effects.

The three-way interaction among cumulative high-altitude residence duration, oxygen supplementation duration, and frequency of returning to low-altitude areas on sleep quality further underscores the complexity of their combined influence on sleep quality. Previous research shows that even after acclimating to high altitudes, individuals residing in these areas continue to experience poorer sleep quality compared to those at lower-altitudes, characterized by reduced slow-wave sleep, lower blood oxygen saturation, and more severe sleep apnea (31, 32). In this study, prolonged high-altitude residence was associated with greater sleep difficulties only under the combined condition of shorter oxygen supplementation sessions and frequent returns to low-altitude areas. This finding suggests that the effect of cumulative high-altitude exposure may not operate independently, but may be amplified when repeated altitude transitions coexist with insufficient oxygen supplementation. Repeated transitions may interrupt stable acclimatization, while shorter oxygen supplementation sessions may be insufficient to maintain adequate oxygenation during sleep or recovery periods. In contrast, either longer oxygen supplementation duration or less frequent altitude transitions appeared to offset some of the risks posed by extended high-altitude residence. Together, these findings highlight the importance of maintaining sufficient oxygen supplementation and managing altitude transition schedules for soldiers deployed for extended periods at high altitudes.

This study has some limitations. First, the cross-sectional design prevents us from drawing causal inferences. Future longitudinal studies are needed to explore the temporal dynamics between lifestyle factors, high-altitude exposure, oxygen supplementation, altitude transitions, and sleep quality among plateau military personnel. Second, the sample consisted predominantly of young male military personnel. Participants were aged 18–45 years, with a mean age of 24.68 years, and only 1.1% were female. Therefore, the findings should be generalized cautiously to female personnel, older adults, civilians, and clinical populations. Third, our reliance on self-reported data may introduce response biases. Future studies should incorporate objective physiological measures (e.g., physiological assessments of sleep quality), third-party assessments, or behavioral observations to further validate the findings. Fourth, although this study assessed oxygen supplementation frequency and duration per session, exact oxygen concentration, oxygen flow rate, and oxygen-supply device type were not collected. Therefore, the dose–response relationship between oxygen supplementation and sleep quality could not be examined. Fifth, frequency of returning to low-altitude areas was measured as an ordinal variable, but the exact reasons for each transition and the detailed duration of each stay in low-altitude areas were not fully captured in the main analyses. Future research should collect more detailed altitude-transition trajectories to better understand repeated acclimatization and de-acclimatization processes.

## 5 Conclusion

This study highlights the combined influence of high-altitude exposure, oxygen supplementation, and altitude-transition patterns on sleep quality among military personnel. The findings suggest that optimizing oxygen supplementation protocols and reducing excessive altitude transitions may serve as practical strategies to improve sleep health and reduce health risks in high-altitude military populations. These results provide important implications for health management and policy development in extreme operational environments.

## Data Availability

The raw data supporting the conclusions of this article will be made available by the authors, without undue reservation.
