# Supplementary material for: Sleep quality among military personnel exposed to high altitude: a cross-sectional study of oxygen supplementation and altitude transition
Source: Front Public Health. 2026 Jun 15;14:1854316. doi: 10.3389/fpubh.2026.1854316 (PMC13311021; doi:10.3389/fpubh.2026.1854316)
Supplement: Supplementary file 1 [file Data_Sheet_1.docx]

**Self-report questionnaire for general information and high-altitude exposure-related characteristics**

The following self-report questionnaire was used to collect participants’ general information and high-altitude exposure-related characteristics. The questionnaire was developed based on previous literature concerning high-altitude exposure, hypoxia-related symptoms, oxygen supplementation, altitude acclimatization, and high-altitude de-acclimatization.

**1. What is your biological sex?**

⚪Male ⚪Female

**2. What is your age?**

____ years

**3. What is your ethnicity?**

⚪Han ⚪Tibetan ⚪Other

**4. Do you have a history of chronic disease?** (Examples of chronic diseases included: chronic obstructive pulmonary disease, asthma, obstructive sleep apnea, hypertension, and other chronic diseases.)

⚪No ⚪Yes

**5. Is this your first time being exposed to a high-altitude environment?**

⚪No ⚪Yes

**6. How long have you lived in high-altitude areas in total?**

⚪ Less than 7 days ⚪7 days-1 month ⚪1-6 months

⚪ 6 months-1 year ⚪ 1-3 years ⚪3-5 years

⚪5-10 years ⚪ More than 10 years

**7. What is the altitude of your current residence?**

⚪2500-3000 m ⚪3000-4000 m ⚪4000-4500 m

⚪4500-5000 m ⚪Above 5000 m

**8. How often do you use oxygen supplementation while staying at high altitude?**

⚪ Never ⚪Occasionally ⚪1-3 times/week

⚪3-5 times/week ⚪Daily

**9. How long do you usually use oxygen supplementation each time?**

⚪Less than 5 minutes ⚪ 5-10 minutes ⚪10-20 minutes

⚪More than 20 minutes

**10. How many times per year do you return to low-altitude areas for vacation or business trips lasting more than 3 consecutive days?**

⚪ None ⚪ 1–2 times/year ⚪ 3–5 times/year

⚪6–9 times/year ⚪ 10 or more times/year

**11. Headache**

⚪ None at all ⚪ A mild headache ⚪ Moderate headache

⚪ Severe headache, incapacitating

**12. Gastrointestinal symptoms**

⚪ Good appetite ⚪ Poor appetite or nausea ⚪ Moderate nausea or vomiting

⚪ Severe nausea and vomiting, incapacitating

**13. Fatigue and/or weakness**

⚪ Not tired or weak ⚪ Mild fatigue/weakness

⚪ Moderate fatigue/weakness ⚪ Severe fatigue/weakness, incapacitating

**14. Dizziness/light-headedness**

⚪ No dizziness/light-headedness ⚪ Mild dizziness/light-headedness

⚪ Moderate dizziness/light-headedness ⚪Severe dizziness/light-headedness, incapacitating
